# Mechanical and Physical Properties of Short Carbon Fiber and Nanofiller-Reinforced Polypropylene Hybrid Nanocomposites

**DOI:** 10.3390/polym12122851

**Published:** 2020-11-29

**Authors:** Harri Junaedi, Muneer Baig, Abdulsattar Dawood, Essam Albahkali, Abdulhakim Almajid

**Affiliations:** 1 Department of Mechanical Engineering, College of Engineering, King Saud University, Po BOX 800, Riyadh 11421, Saudi Arabia; ebahkali@ksu.edu.sa (E.A.); aalmajid@ksu.edu.sa (A.A.); 2 Department of Engineering Management, College of Engineering, Prince Sultan University, Po BOX 66833, Riyadh 11586, Saudi Arabia; mbaig@psu.edu.sa; 3 Saudi Arabian Basic Industries Corporation (SABIC), Po BOX 5101, Riyadh 11422, Saudi Arabia; abdulsattar@sabic.com

**Keywords:** polypropylene, hybrid, composite, polymer, short carbon fiber, nanoparticles

## Abstract

The effect of various combinations of filler materials on the performance of polypropylene (PP)-based composites was investigated. PP in particulate form was used as the matrix. Milled short carbon fiber (SCF) micro-size, graphite nano-platelet (GNP), and titanium dioxide nanoparticles (nTiO_2_) were used as fillers. These fillers were incorporated in the polymer matrix to produce mono-filler (PP/SCF and PP/nanofiller) and hybrid composites. Hybrid composites consist of PP/10SCF/GNP, PP/10SCF/nTiO_2_, and PP/10SCF/GNP/nTiO_2_. The effect of the addition of SCF, GNP, and nTiO_2_ on PP-based composites was investigated by analyzing their morphological, mechanical, and physical properties. The addition of mono-filler to the PP matrix improved the mechanical properties of the composites when compared to the neat PP. The ultimate tensile strength (UTS), flexural modulus, flexural strength, and impact toughness of the hybrid composites with 15 wt % total loading of fillers, were higher than that of mono-filler composites with 15 wt % SCF (PP/15SCF). A maximum increase of 20% in the flexural modulus was observed in the hybrid composite with 10 wt % of SCF with the additional of 2.5 wt % GNP and 2.5 wt % nTiO_2_ when compared to PP/15SCF composite. The addition of 2.5 wt % nTiO_2_ to the 10 wt % SCF reinforced PP, resulted in increasing the strain at break by 15% when compared to the PP/10SCF composite. A scanning electron microscope image of the PP/10SCF composite with the addition of GNP improved the interfacial bonding between PP and SCF compared with PP/SCF alone. A decrease in the melt flow index (MFI) was observed for all compositions. However, hybrid composites showed a higher decrease in MFI.

## 1. Introduction

Thermoplastics are very common materials that are used extensively in our daily lives, such as in plastic bags, bottles, and containers. Thermoplastic materials have limited uses because of their relatively weak mechanical and physical properties. The performance of thermoplastics also tends to decrease with time due to environmental effects and loading conditions [1]. Consequently, thermoplastic materials are used heavily in non-load-bearing applications, such as in the automotive industry i.e., car bumper, door panel, engine cover, dashboard, etc., due to their lightweight, processability, and recyclability [2]. Several studies have been conducted to investigate the enhancement of the mechanical and physical properties of thermoplastic polymer materials. Fibers or fillers in the form of polymer composites are often used to strengthen and improve the properties of polymers. Continuous fiber reinforced polymer composites [3], long fiber reinforced polymer composites [4,5,6], short-fiber reinforced polymers composites [5,7], nanofiller-reinforced polymer composites [8,9,10,11,12], and hybrid composites, which are a combination of these composites [13,14,15] are some of the compounding strategies used to improve the properties of polymers.

Unlike thermoset plastics, which can easily be combined with continuous fiber to produce large parts, there are limitations to the combination of continuous fiber and thermoplastic [16]. Long- and short-fiber reinforced thermoplastic composites are widely used. When the fiber length to diameter ratio is within the range of 100, the fiber is called short-fiber [17]. The advantage of short fibers compared to continuous or long fiber, in combination with thermoplastic polymers, is their processability. Nevertheless, short-fiber reinforced polymer composites have weak mechanical properties.

Fillers are solid particulates of different shapes and inorganic and organic materials that are used in plastics to modify their properties. Mechanical, fire retardant, electrical, magnetic, surface, and processing properties can be modified through the addition of fillers. Fillers can also be used for enhancing degradability and antiaging, and as gas barriers and warpage reducers [18]. Different structural, shape, surface, mechanical, and physical properties of fillers influence the overall properties of thermoplastic composites [19]. Some studies show that adding spherical shapes, such as silica nanoparticles, improves toughness and elasticity [20], while the addition of similar particles in fiber shape increases the modulus of elasticity and strength [16]. Recently, the combination of micro-scale and nano-scale fillers has become an area of significant interest. The use of a small number of nanofillers is expected to increase the interfacial shear stress between the matrix and macro-filler, leading to an improvement in both the mechanical and physical properties of the composites [13]. Arao et al. [13] combined short carbon fiber/polypropylene composites with different types of nanofillers, such as silica, alumina, carbon nanotube (CNT), and nano-clay. They concluded that the addition of nanofillers and maleic anhydride increases the interfacial shear strength between polypropylene and carbon fibers by up to 60%, with the exception of nano-clay. Furthermore, the addition of nanofillers helps improve the strength, modulus, and crack resistance of the matrix.

Thermoplastic materials, such as polypropylene, polyamide 6, polyamide 66, polyethylene, polycarbonate, nylon, and Acrylonitrile butadiene styrene (ABS), are used heavily in the automotive industry and for other applications. Micro-scale materials, such as short carbon fiber (SCF) and glass fiber [7], and nanofillers, such as graphite nano-platelet (GNP) [21], graphene oxide [22], CNT [23], SiO_2_ nano-sphere [20,24,25], and TiO_2_ nano-sphere [26], can be used as fillers in thermoplastic composites. Incorporating nanofiber into polymers is also an area of interest for researchers [27,28].

The processing of micro and nanofiller-reinforced thermoplastic composites was carried out by using conventional methods. Microfiller, nanofiller, and thermoplastic materials are pre-mixed in different percentages to disperse the filler into the thermoplastic matrix. Thermoplastic composites with varying filler materials can be melt-mixed using a twin-screw extruder to produce pellets of composite material that are later injection-molded to produce a thermoplastic composite component.

The novelty of this work is to study synergistic responses of the binary and ternary filler materials in polypropylene (PP)-matrix composites. For this purpose, PP was reinforced with short carbon fiber (SCF), graphite nano-platelet, and TiO_2_ nanoparticles to produce hybrid polymer nanocomposites. The PP used was in the form of particulates instead of pellets. A mechanical stirring process was used in the pre-mixed stage to increase the dispersibility of the filler in the PP matrix. The introduction of a second nanoparticle might improve the dispersion of the nanoparticles as previously has been reported by Yang et al. [29] and Chatterjee et al. [30]. Thus, in addition to micro-size short carbon fiber (SCF), GNP, and titanium dioxide nanoparticles (nTiO_2_) were added to create a hybrid nanocomposite. Tensile tests, flexural tests, and impact experiments were performed to investigate the mechanical properties of the composites. Meanwhile, the density and melt flow index (MFI) were measured to observe the change in the physical properties of the composites. Morphology and fractography analyses were also performed on the fabricated composites.

## 2. Materials and Methods

### 2.1. Materials

PP homopolymer in the form of particulates was obtained from the Saudi Arabian Basic Industries Corporation (SABIC), Riyadh, Saudi Arabia and used as the matrix. The MFI of the PP was 22.7 g/10 min at a temperature of 230 °C and load of 2.16 kg. Particulate PP was used instead of pellets to enhance the dispersion of fillers in the matrix through mechanical mixing. The average diameter size of the particulate PP was 560 µm. Irganox 1010, Irgafos 168, and calcium stearate were used as additives to prevent degradation in the PP during processing. Irganox 1010 and Irgafos 168 were supplied by BASF, Ludwigshafen, Germany and used as anti-oxidants, while calcium stearate was used as a free radical scavenger. An SCF with a diameter of 7–9 µm and an average length of 150 µm was supplied from Asbury Carbon Inc., Asbury, NJ, USA. The SCF was made from polyacrylonitrile (PAN) fiber with a carbon content above 94% and un-sized surface. GNP grade Nano307 with a surface area of 325–375 m^2^/g, diameter <1 µm, thickness of ~3 nm, and a true density of 2.16 g/cm^3^ was supplied from Asbury Carbons Inc., Asbury, NJ, USA. Rutile grade TiO_2_ nanoparticles (nTiO_2_) with an average diameter of 30 nm were supplied from US Research Nanomaterials, Inc., Houston, TX, USA. All the other properties of the raw materials used in this study are presented in Table 1.

The PP/SCF, PP/nanofillers, PP/SCF/nanofillers were weighed as per the composition presented in Table 2. Irganox 1010, Irgafos 168, and calcium stearate were added to all compositions at a fixed amount of 0.08, 0.1 and 0.05 wt %, respectively. They were first pre-mixed using a mechanical mixer with speed of 500 rpm for 15 min at room temperature to disperse the carbon fiber and/or the nanofillers into the PP matrix. Then, they were taken to the twin-extruder machine. The pre-mixed raw materials were extruded at a fixed temperatures which has ten different zones within the range 160 to 210 °C and a speed of 50 rpm to produce a strand and later fed into the pelletizer to produce pellets. The screw diameter of the twin-screw extruder was 16 mm with a 40 L/D (length to diameter ratio). The composite pellets were later introduced into an injection molding machine whose barrel temperatures was with three different zones which are 180, 195, and 220 °C to produce testing samples of the composites.

### 2.2. Methods

#### 2.2.1. Characterization of Fillers

A scanning electron microscope (SEM) was used to characterize the morphology of the fillers. The SCF length was received and calculated after processing using SEM by averaging the measurements for 100 fibers. The composite samples were calcinated in a furnace in a nitrogen atmosphere to 550 °C to examine the SCF length after processing. At this temperature, the PP matrix was evaporated, leaving behind the fillers, which were analyzed using SEM to measure the length of fibers after processing.

#### 2.2.2. Melt Flow Index Measurement

MFI is a measure of the ability of the material to flow under certain temperature and pressure conditions. The MFI has an inverse correlation with molecular weight, and lower MFI correlates with higher molecular weight [31]. An MFI test was conducted to investigate the influence of carbon fiber/fillers on the MFI of the composites. The test was conducted following American Society for Testing and Materials (ASTM) standard D1238 [32] at a temperature of 230 °C and load of 2.16 kg. Procedure A from the standard was used to measure the melt flow rate. It was based on the mass of polymer that extrudes from a specific die dimension over a specific time. The MFI unit is the grams of material per 10 min (g/10 min).

#### 2.2.3. Density Measurement

The addition of fillers into the polymer matrix changes the density of the composite compared with the neat polymer. The actual density of the neat PP and its composite was measured using a densitometer device based on the Archimedes principle. The actual density was calculated using Equation (1). Isopropyl alcohol (IPA) with a density of 0.786 g/cm^3^ was selected as the liquid instead of water since the PP has a lower density than water.

*ρ*_a_ = *ρ_w_W*_a_/(*W*_a_−*W*_m_) (1)

where *ρ*_a_ is the actual density of the composites, *ρ*_m_ is the density of isopropyl alcohol, *w_a_* is the weight of the sample in air, and *W*_m_ is the weight of the sample in the IPA medium. The theoretical density (*ρ*_t_) of the composites was calculated by using an equation developed by Agarwal et al. [33] shown in Equation (2). In this equation, *w*_f1_, *w*_f2_, and *w*_f3_ are the weight fraction for carbon fiber/nanofillers, *w*_m_ is the weight fraction for matrix, *ρ*_f_ is the density of carbon fiber/fillers and, *ρ*_m_ is the density of the matrix. The densities of PP, SCF, GNP, and TiO_2_ that were used were 0.9 g/cm^3^, 1.79 g/cm^3^, 2.16 g/cm^3^, and 4.23 g/cm^3^, respectively.

*ρ*_t_ = 1/(*w_f_*_1_/*ρ_f_*_1_ + *w_f_*_2_/*ρ_f_*_2_ + *w_f_*_3_/*ρ_f_*_3_ + *w_m_*/*ρ_m_*) (2)

#### 2.2.4. Morphology and Fractography of Composites

An optical microscope (OM) and SEM were used to characterize the microstructure of the composites. The SEM was also used to observe the tensile and impact fracture surface of the specimens. For SEM purposes, the surface of samples was coated with platinum before observation.

#### 2.2.5. Tensile Test

**Figure 1:**
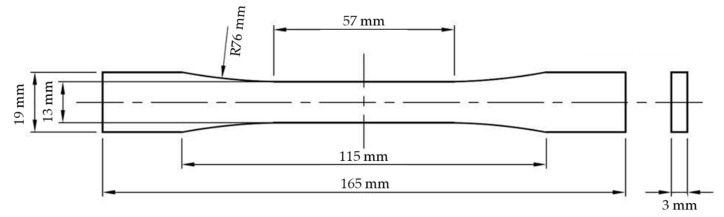
Tensile test specimen dimension according to American Society for Testing and Materials (ASTM) D638 Type I [34].

Uniaxial tensile experiments were performed on the composites following ASTM D638 [34] to measure the modulus of elasticity, UTS, and strain at break. Figure 1 shows the dimension of the testing sample that was manufactured in accordance with ASTM D638 Type I. The experiments were performed at room temperature using an Instron Universal Testing Machine. The cross-head speed of the testing machine was 5 mm/min. The strain values in the elastic region were measured using an extensometer. The modulus of elasticity was calculated using the stress versus stress in the elastic region. At least three tensile samples were tested for each composition.

#### 2.2.6. Flexural Test

Flexural tests were conducted using a Universal Testing Machine (UTM) INSTRON 3385H following the International Standard ASTM D790 [35] using a standard three-point bending fixture. Three specimens were tested for each composition. The support span (L) was set to 52 mm, and the cross-head speed was 1.5 mm/min.

#### 2.2.7. Impact Test

Notched impact tests were conducted using an Izod impact testing machine to measure the energy that can be absorbed during impact load as per ASTM D256 [36]. The maximum impact energy was 5.5 J, and the impact speed was 3.5 m/s. Three specimens were tested for each composition.

## 3. Results and Discussion

### 3.1. Fillers Characterization

**Figure 2:**
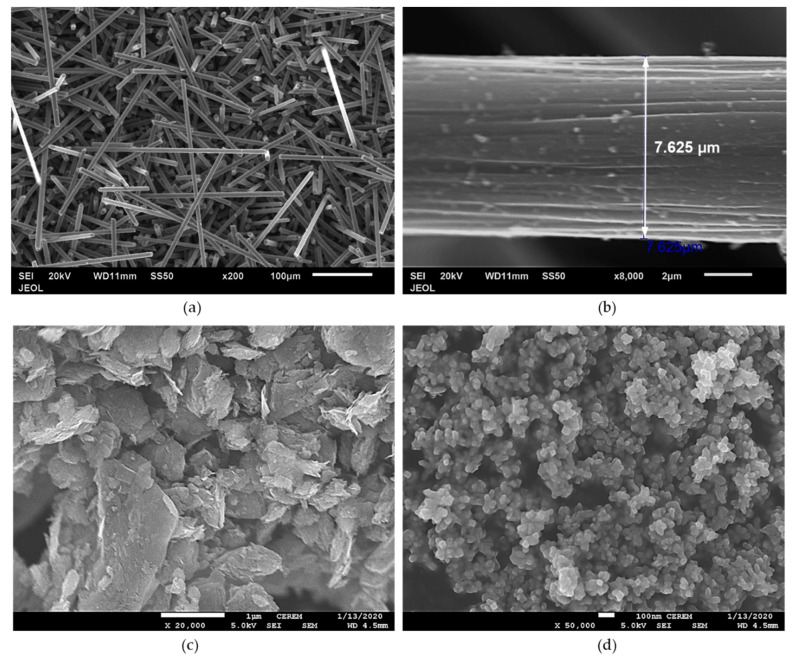
SEM images of raw materials (**a**,**b**) short carbon fiber at different magnifications, (**c**) graphite nano-platelet, (**d**) titanium dioxide nanoparticles.

Figure 2a,b shows the morphology of SCFs. Figure 2a shows that most of the fibers have length >100 µm, and the enlarged picture, Figure 2b, shows that the diameter of the fiber was ~7 µm, and the surface was un-sized. The average length of the fiber from the measurement was 150 µm, as mention by the manufacturer. Figure 2c shows the morphology of GNP. It is evident that the diameter of the GNP was below 1 µm. Meanwhile, the thickness of the GNP was on the nano-size scale. Figure 2d shows the morphology of the nTiO_2_. The diameter of most of the particles was less than 30 nm, as stated by the manufacturer. The characterization of the as-received filler was in agreement with the manufacturer specifications.

Table 3 presents the surface area of the fillers. The surface areas of SCF and nTiO_2_ were obtained from the known dimensions of the filler. The SCF had a diameter of 7 µm, a length of 150 µm, and a density of 1.79 g/cm^3^, while the nTiO_2_ had a spherical shape with a diameter of 30 nm and density of 4.23 g/cm^3^. The surface area/weight of GNP was taken from the supplier datasheet. Meanwhile, the surface area/volume was calculated by multiplying the surface area/weight by its density (2.16 g/cm^3^)_._ It was noticed that the GNPs had the highest surface area among the three fillers.

Table 4 shows a comparison of fiber length of as-received fibers and fibers after processing for PP/SCF composite and PP/10SCF/Nanofiller hybrid composites. The average fiber length of as-received SCF was 150 µm, in accordance with the specification from the manufacturer. In general, a decrease in fiber length was observed on all composites compared with as-received SCF. The manufacturing processes, such as mixing, extrusion, and injection molding, can lead to fiber breakage [37]. PP/10SCF/5GNP and PP/10SCF/2.5GNP/2.5nTiO_2_ hybrid composites exhibit a greater reduction in average fiber length compared with the PP/10SCF composite. Meanwhile, for PP/10SCF/5nTiO_2_, almost no reduction in fiber length was observed compared with that of PP/10SCF. It was observed that the addition of GNPs contributed to the reduction in the average fiber length after processing. This could be due to decreases in the MFI (as shown in Table 5) or increases in the viscosity through the addition of GNP. The increase in viscosity might have led to an increase in the shear forces during processing. Higher shear forces might be attributed to the increase in fibers breakage [38].

### 3.2. Melt Flow Index Measurement

The MFI of the neat PP was 22.7 g/10 min at a temperature of 230 °C and load of 2.16 kg. The MFI of all composites decreased with the addition of carbon fiber or nanofillers. The decrease in MFI by the addition of nanofillers also has been reported by others [39,40]. The addition of filler on the polymer matrix has lowered the mobility of the polymer molecule chains [40]. At 5 wt % loading of different fillers, the highest reduction in MFI occurred in the GNP composite, where MFI reached 6.2 g/10 min. Additionally, the PP/10SCF/Nanofiller hybrid composite, at 10 wt % loading of SCF, the MFI decreased with the addition of wt % of nanofillers. The maximum reduction in MFI was at 5 wt % of GNP, where MFI reached 4.4 g/10 min. GNP had the highest degree of interaction with the PP molecule chain. This implies that the GNP distribution and dispersion was better than the dispersion of nTiO_2_ and SCF, which could be ascribed to the high surface area of the GNP compared with the other two fillers (Table 3). Even at 1 wt % of GNP loading, the MFI was lower than 20 wt % of SCF loading. The hybrid composites showed the same trend as the composites of the precursor filler.

### 3.3. Density

The densities of neat PP and PP composites are presented in Table 5. The measured densities are in agreement with the theoretical density. The measured and theoretical densities are equivalent when the voids in the composites are minimal and filler wt % loading conforms with the pre-scribed compositions. The addition of nanofillers often introduces voids on the composite because of the agglomeration of the filler materials. However, in these composites, this problem did not exist or was minimal. PP has a lower density than SCF, GNP, and TiO_2._ Therefore, the addition of these fillers increased the density of the composites compared with neat PP.

### 3.4. Microstructures

**Figure 3:**
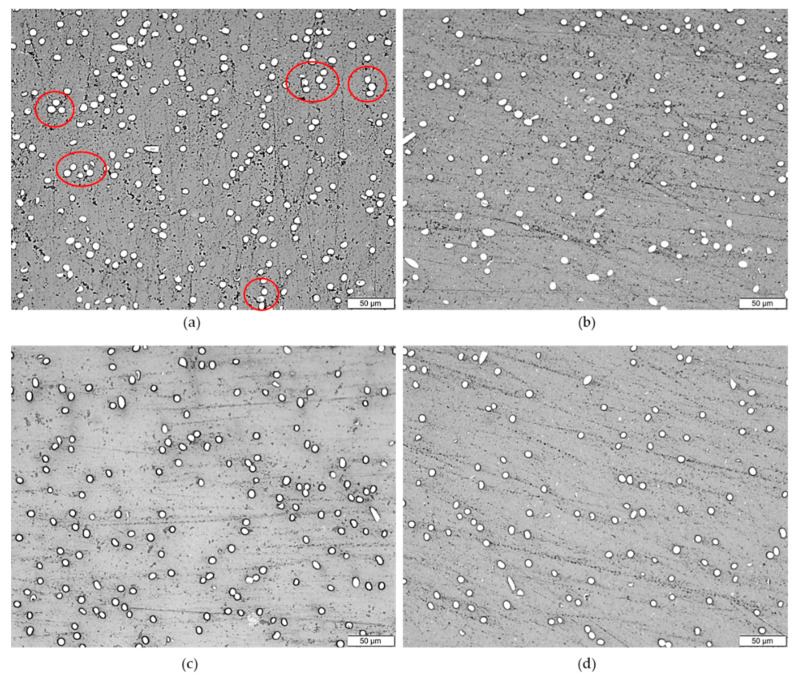
Optical microscope images of cross-section surfaces of (**a**) PP/15SCF, (**b**) PP/10SCF/5 nTiO_2_, (**c**) PP/10SCF/5GNP, and (**d**) PP/10SCF/2.5GNP/2.5nTiO_2_ composites.

**Figure 4:**
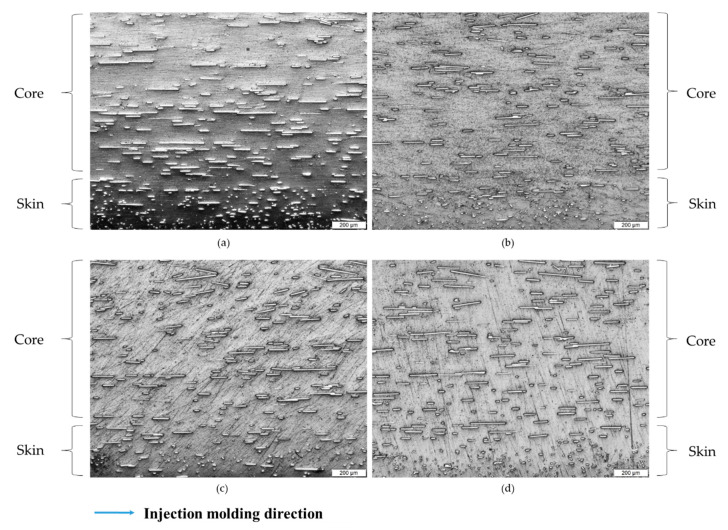
Optical microscope images of longitudinal section surfaces of (**a**) PP/15SCF, (**b**) PP/10SCF/5nTiO_2_, (**c**) PP/10SCF/5GNP, and (**d**) PP/10SCF/2.5GNP/2.5nTiO_2_ composites.

Figure 3 shows cross-section surfaces of different composite compositions at 15 wt % loading of filler using an OM. Figure 3a shows the composite with 15 wt % SCF loading and that some fibers already start to form an agglomerate, marked by red circles. At 10 wt % SCF loading with the addition of nanofillers (Figure 3b–d), the agglomeration was observed to be minimal. Figure 4 shows the longitudinal section of the same composites. The injection molding direction is shown by the arrow on the bottom of the figure. The microstructure images show different skin and core structures. For all microstructures, fibers in the core of the samples tend to align with the direction of the injection molding while the fibers in the skin of the samples are oriented perpendicular to the inject direction. The different structures in the skin and core of the injected molding fiber reinforced polymer have been also reported previously by others [41,42].

**Figure 5:**
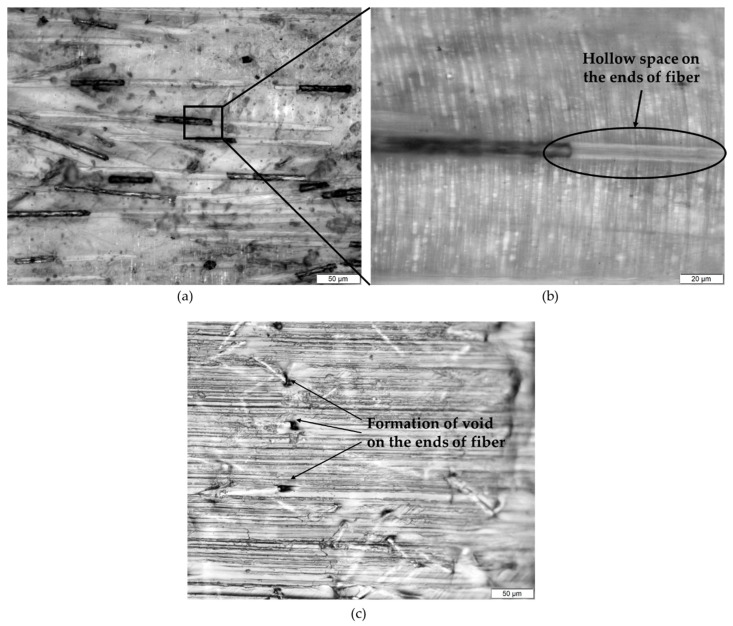
Optical microscope images of (**a**) PP/5SCF, (**b**) image in panel (**a**) at higher magnification, and (**c**) PP/10SCF composite after tensile test.

The microstructure of PP/5SCF composite for the sample that was deformed under tensile loading is shown in Figure 5a,b. As shown in the figure, the PP/5SCF composite experiences very high strain of up to 450%. The microstructure showed a hollow space at the ends of the fibers due to the elongation of the matrix. Figure 5c shows a PP/10SCF composite surface after the tensile experiment. It shows that microvoids were generated at the ends of the fiber without further elongation due to failure, as shown in Figure 5a,b. The microvoid is most probably the starting point for the formation of the hollow space that was observed in Figure 5a,b in PP/5SCF composites. Stress concentration generated at the fiber end due to debonding between the PP matrix and the fiber may be responsible for this phenomenon.

**Figure 6:**
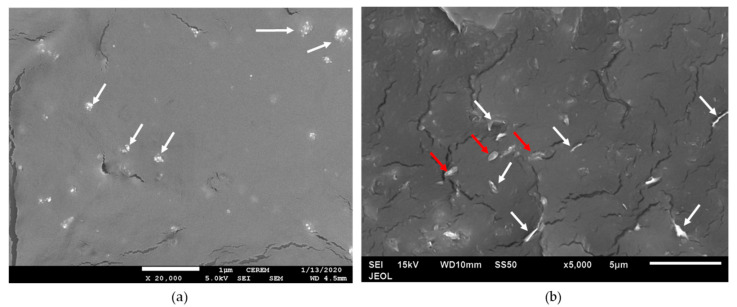
SEM images of microstructures of (**a**) PP/10CF/nTiO_2_, and (**b**) PP/10CF/5GNP.

Figure 6 shows the nanofillers GNP and nTiO_2_ in PP/10SCF/5GNP and PP/10SCF/5nTiO_2_ composites. Figure 6a shows the microstructure of the PP/10SCF/5nTiO_2_ composite. Agglomeration of nTiO_2_ was observed (marked by the white arrow). However, most of the agglomerations were below 1 µm and embedded strongly to the PP matrix. In general, nTiO_2_ nanoparticles were not fully dispersed. The mechanical stirring process used for dispersing the nTiO_2_ in particulate PP was not able to fully break down the strong aggregate of nTiO_2_. Figure 6b shows the microstructure of the PP/10CF/5GNP composite. Single GNPs oriented perpendicular (marked by the white arrow) and parallel (marked by the red arrow) to the surface were observed as embedded in the PP matrix. In general, the dispersion of GNP in the PP matrix was better than that of nTiO_2_.

**Figure 7:**
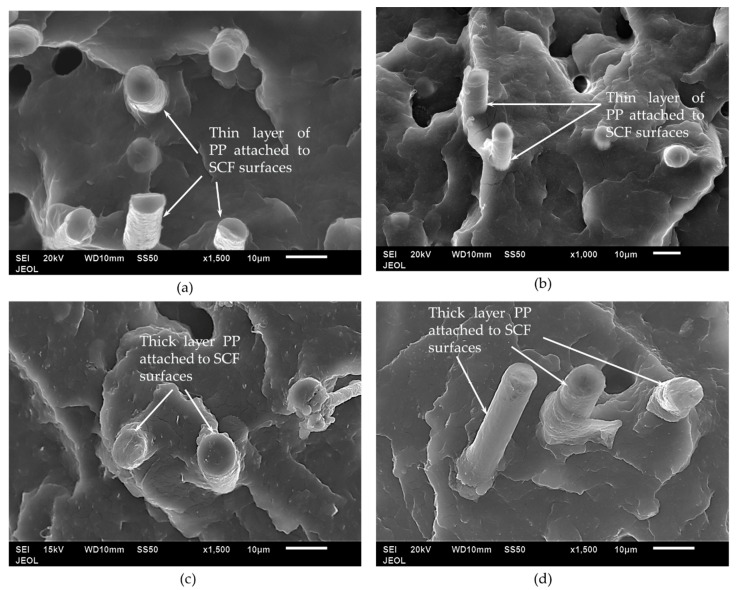
SEM images microstructures of (**a**) PP/15SCF, (**b**) PP/10SCF/5nTiO_2_, (**c**) PP/10SCF/5GNP, and (**d**) PP/10SCF/2.5GNP/2.5nTiO_2_ composites.

Figure 7 shows the microstructure of different composites at 15 wt % loading. All composites show strong bonding between SCF and PP. From the figure, it is evident that SCF was pulled out from the matrix during the tensile test. The presence of GNP increases the interfacial shear strength between the SCF and PP matrix, as indicated by the thick PP layer that was attached to the SCF surface seen in Figure 7c,d, compared with the thin layer of PP on the SCF surface of PP/10SCF and PP/10SCF/5nTiO_2_ composites (Figure 7a,b). This conclusion is confirmed by the tensile strength data of the composites, which will be discussed later. Arao et al. [13] showed that the interfacial shear strength between the fiber and matrix improved with the addition of nanofillers. Sapiai et al. [43] also reported the improvement of interfacial bonding between the polymer matrix and the fiber with the addition of nanofillers.

### 3.5. Tensile Test Fracture Surfaces

**Figure 8:**
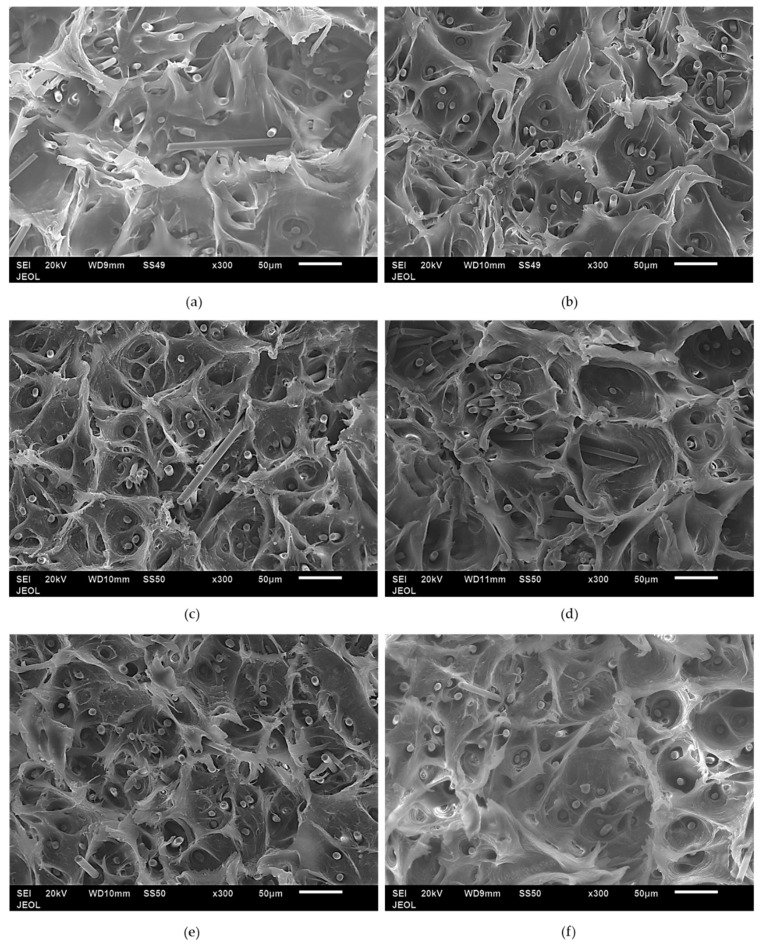
SEM images of tensile test fracture surfaces (**a**) PP/10SCF, (**b**) PP/15SCF, (**c**) PP/10SCF/5GNP, (**d**)PP/10SCF/5nTiO_2_, (**e**) PP/10SCF/4GNP/1nTiO_2_ and (**f**) PP/10SCF/2.5GNP/2.5nTiO_2_.

Figure 8 shows the fracture surfaces of tensile test samples. The uniaxial loading direction was perpendicular to the fracture surface. The fracture surfaces for Figure 8a–e show ductile fractures with dimples and fibrillar structure. The dimple and fibrillar structures are created from the microvoid, which is formed during the elongation of the composite since SCF has a very low ductility, while the PP matrix has high ductility. This results in the formation of microvoids at the end of the fiber, which continue to elongate as the composites undergo tensile loading, as shown in Figure 5, at low loading (PP/5SCF). At higher loading, the fiber content increases, and the fibers reach a close proximity with each other. Microvoids on each fiber end coalesce to form a bigger void, leading to failure. Additionally, the figure shows that some of the fibers are oriented perpendicularly or are misaligned to the loading direction. The combination of fiber ends and fibers that are misaligned with the loading direction could be responsible for the fracture mechanism observed in the composites.

### 3.6. Tensile Test

The mechanical properties from the tensile experiments are presented in Table 6. In general, the addition of carbon fiber or nanofillers increased the tensile modulus and UTS of the composites for all combinations considered in this study. The increase in tensile strength of all types of fillers indicates that the use of particulate PP along with the mechanical stirrer and twin-extruder was effective to disperse the fiber and nanofiller material within the PP matrix.

**Figure 9:**
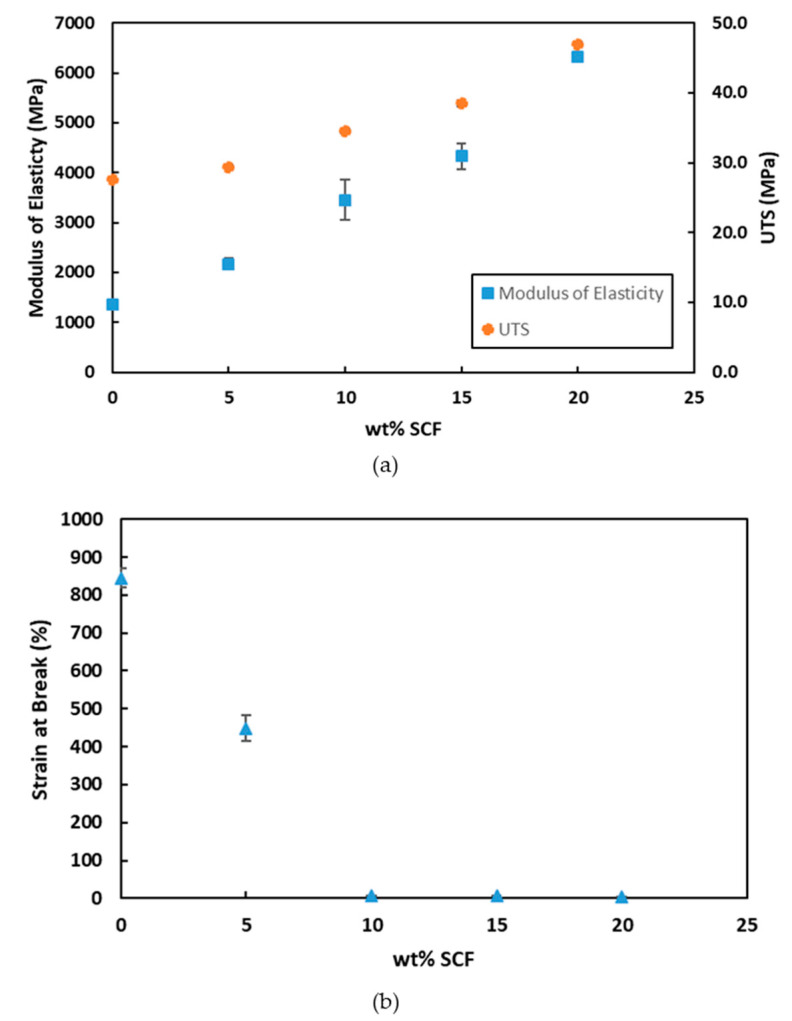
Mechanical properties of PP/SCF composites (**a**) modulus of elasticity and ultimate tensile strength (UTS) and (**b**) strain at break vs. wt % SCF.

Figure 9 shows the mechanical properties of PP/SCF composites. For PP/SCF composites up to 20 wt % SCF loading, the tensile modulus increased up to 6314 MPa, which corresponds to an increase of 365% from the neat PP. Similarly, the UTS increased by 70% from 27.6 to 46.9 MPa. Meanwhile, the strain at break decreased from 845% to only 5.3%. Similar behavior was reported earlier by other researchers [44,45]. An increase in the tensile modulus and tensile strength could be attributed to the load transfer from the PP matrix to SCF. This is because carbon fiber has a very high strength and modulus and very low ductility. During the manufacturing process, fibers can break into smaller short fibers, due to the shear force developed by extrusion machine screws and fiber to fiber interaction [7,37]. The results obtained in this study and shown in Table 4 verify this condition. A shorter fiber creates more fiber ends. These fibers end and fiber with perpendicular direction with the tension loading would be a preferable location for the failure path. At 10 wt % of loading, carbon fibers are in close proximity with each other, and in some places, they start to create agglomerations. These agglomerations can create a stress concentration at the ends of the fibers and result in the formation of voids that might be responsible for reducing the strain at break, that has been explained before, on the tensile test fracture surface subsection.

**Figure 10:**
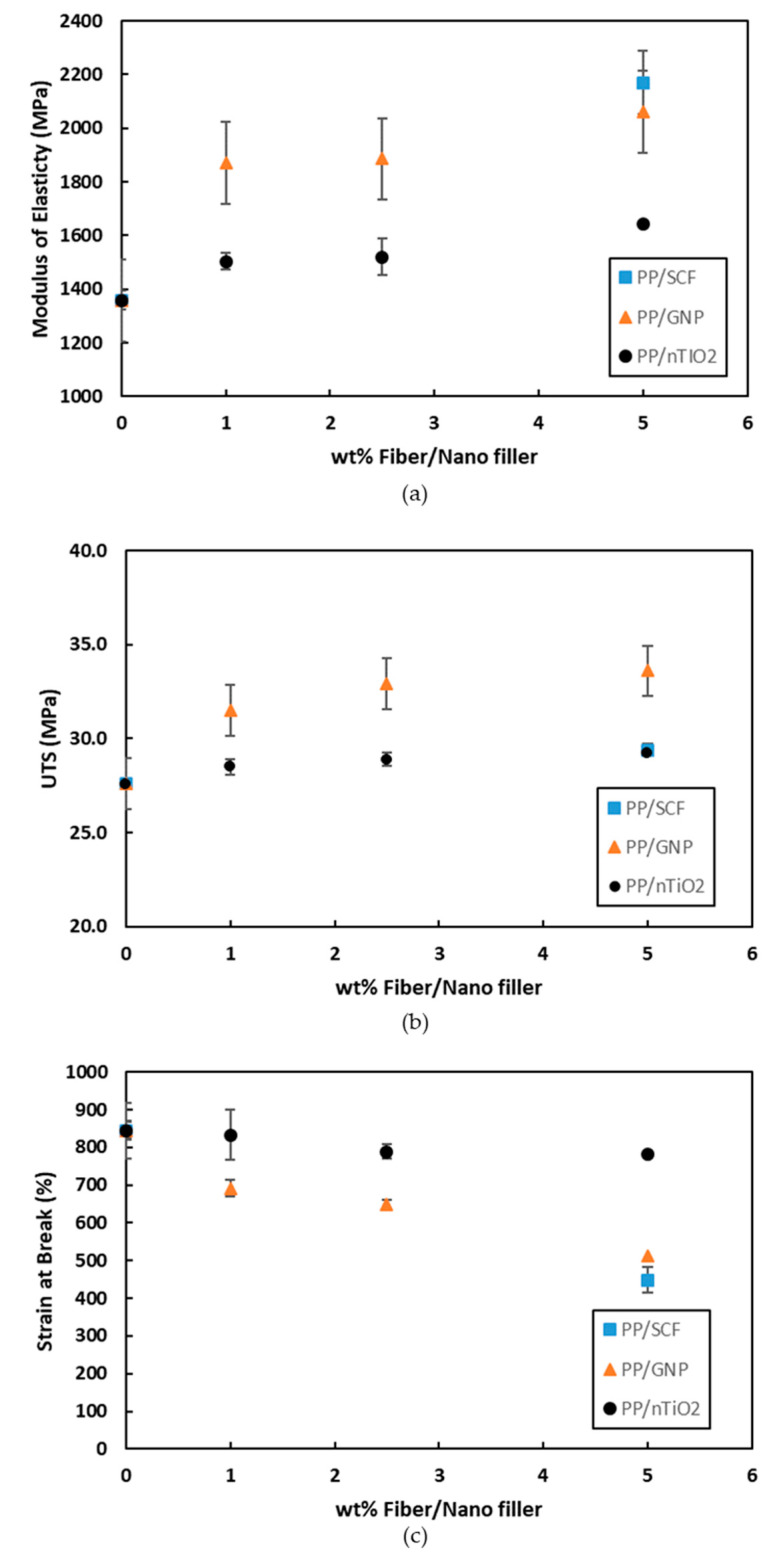
Mechanical properties of PP composites with different fillers (**a**) Tensile modulus, (**b**) Ultimate tensile strength (UTS), and (**c**) Strain at break.

The comparison of modulus of elasticity, UTS, and strain at break for composites of different fillers as a function of filler weight fraction is presented in Figure 10a–c. For the PP/GNP and PP/nTiO_2_ composite systems, up to 5 wt % loading of nanofillers, the maximum tensile modulus was shown by PP/5GNP. The tensile modulus increased by 58% and was also followed by increases in the UTS from 27.6 to 33.6 MPa, while retaining the strain at break to 514%. When comparing the three different types of fillers at 5 wt % loading, GNP showed a higher ability to affect the UTS compared with the SCF and nTiO_2_, as presented in Figure 10b. From the characterization of fillers (Table 3) it is evident that GNP has the largest surface area of the three fillers. The higher surface area increases the interaction between the GNP and PP chain molecules. This interaction consequently leads to inhibition of the movement of PP chain molecules and stress transfer from the matrix to GNP. It has been previously reported, that GNPs can also increase the crystallinity degree of the polymer [46], implying that they also increase the strength of the polymer. Meanwhile, for PP/GNP/nTiO_2_ composites at 5 wt % total loading, the properties reasonably lay between PP/5GNP and PP/5nTiO_2_.

**Figure 11:**
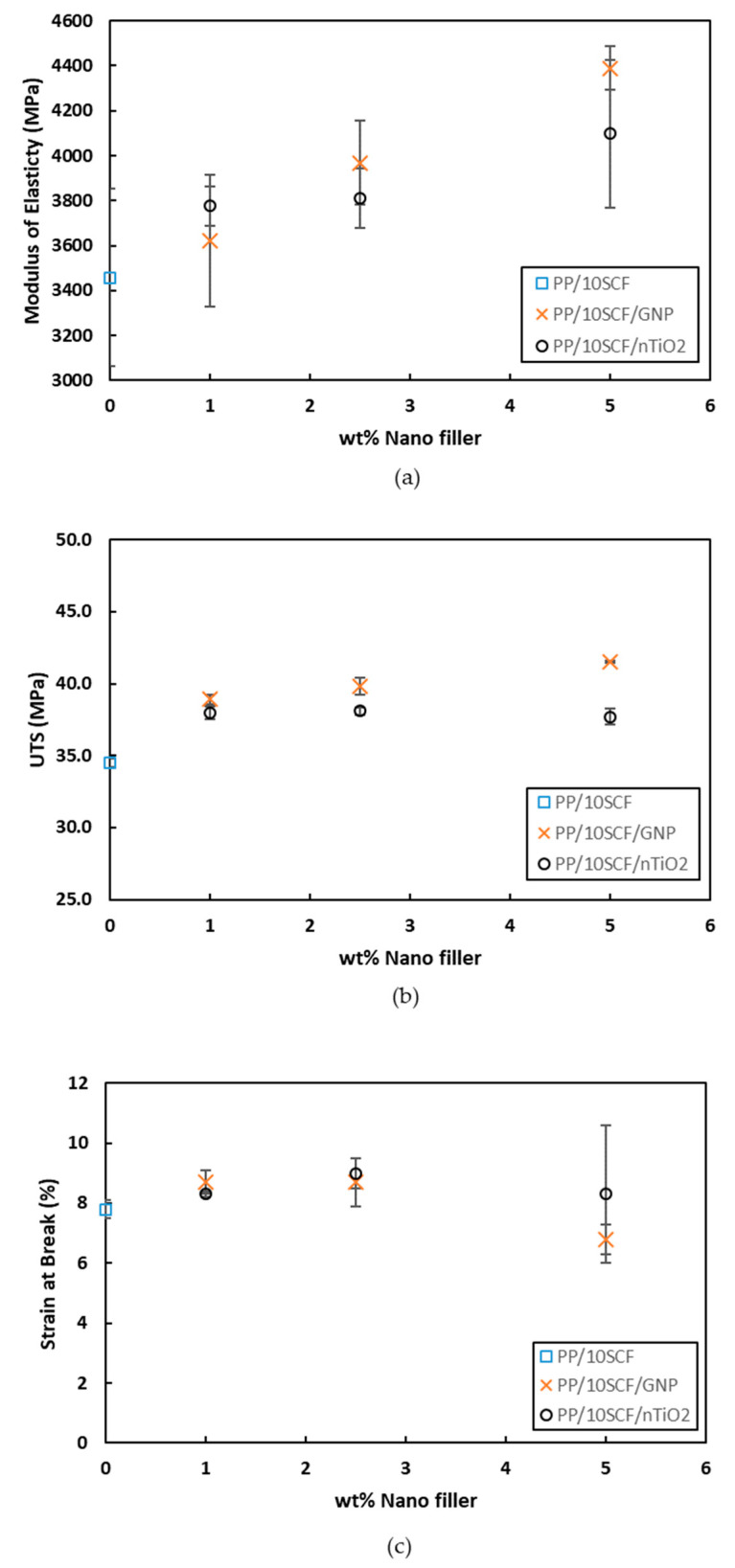
(**a**) Tensile modulus, (**b**) Ultimate tensile strength (UTS), and (**c**) Strain at break vs. wt % PP/10SCF/nanofiller.

Figure 11 presents the modulus of elasticity, UTS, and strain at break for PP/10SCF/nanofiller for different nanofillers at different wt % loading. The same tendencies as the PP/nanofiller were found for these composites. PP/10SCF/GNP composites exhibit a maximum tensile modulus and UTS at 5 wt % of GNP, which increased by ~27% and ~20%, respectively, compared with the PP/10SCF composite (Figure 11a,b). Strain at break also improved from 7.8% for the PP/10SCF composite, to 8.7% for the PP/10SCF/2.5GNP composite (Figure 11c). An increase in ductility, plausibility due to the bridging effect of GNP, was perpendicular to the tensile load direction [21]. Meanwhile, the decrease in the ductility by the addition of GNP was due to the increase in agglomeration of GNP and the orientation of GNP that were parallel to the tensile load direction [47]. PP/10SCF/nTiO_2_ composites also showed an increase of up to 18.5% in the tensile modulus at 5 wt % nTiO_2_ and an increase in UTS of up to 10% at 2.5 wt % nTiO_2_ compared with the PP/10SCF composite. Then, the UTS decreased at 5 wt % of nTiO_2_. The reduction in UTS at 5 wt % of nTiO_2_ was attributed to the agglomeration of nTiO_2_. Strain at break improved from 7.8% for the PP/10SCF composite to 9% for the PP/10SCF/2.5nTiO_2_ composite, as presented in Figure 11c. The agglomeration of a spherical nanoparticle could lead to crack blunting [48] or debonding of nanoparticle agglomerates, which leads to plastic void growth [49,50]. In general, the addition of GNP is more effective in improving the tensile modulus and UTS of the composites, while the presence of spherical nTiO_2_ is more effective in improving the strain at break. The improvement of toughness and ductility on fiber-reinforced polymer by the addition of spherical shape nanoparticles at certain content has been reported also by Rasana and Jayanarayana [51] and Rasana et al. [52].

**Figure 12:**
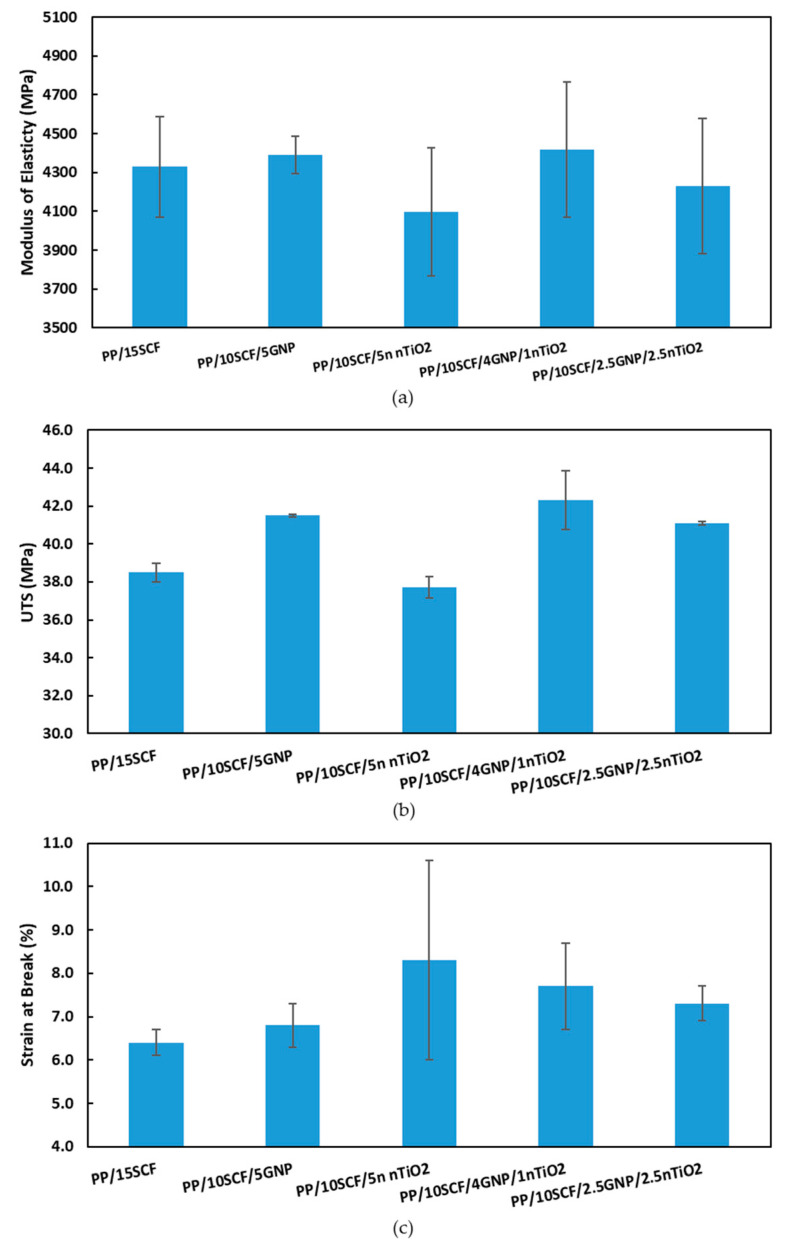
(**a**) Tensile modulus, (**b**) ultimate tensile strength (UTS), and (**c**) strain at break at 15 wt % loading for different composites.

A comparison of tensile modulus, UTS, and strain at break values of the composites at 15 wt % loading with different filler combinations is presented in Figure 12. The PP/10SCF/4GNP/1nTiO_2_ composite showed better properties compared with other compositions at the same wt % loading. The properties were still lower than those of 20 wt % of SCF. The use of different types of fillers increased the tensile modulus and UTS up to 226% and 53%, respectively, compared with the neat PP, as shown in Figure 12a,b. However, if we compare the PP/10SCF/4GNP/1nTiO_2_ composite with PP/15SCF, the increase in the UTS of the hybrid composite was almost 10%, while there was almost no increase in the tensile modulus. The reason for the increase in strength was attributed to an improvement in the dispersion of the fillers through the presence of different types of fillers. Yang et al. [29] and Chatterjee et al. [30] also used the combination of two different nanofillers to increase the dispersion of nanofillers. However, the strain at break increased to 7.7% of strain, which was an increase of 20% compared with the PP/15SCF composite (Figure 12c).

### 3.7. Flexural Test

Table 7 shows the flexural test results of PP and its composites. The flexural test results agreed with the tensile test results. For the PP/SCF composite system, the increase in SCF loading up to 20 wt % resulted in an increase in the flexural modulus and flexural strength up to 240% and 100%, respectively. However, the increase in flexural strength was higher when compared with the increase in the UTS of the composite. This occurred because the stress distribution in the flexural test was not uniform, and it occurred across a small volume compared with the tensile test in which uniform stress occurred across a larger volume. The same behavior was reported by Din and Hashemi [53].

For the PP/GNP and PP/nTiO_2_ composites at up to 5 wt % loading of nanofillers, there were similar results to those for tensile strength: the maximum flexural modulus and flexural strength were increased by 60% and 31%, respectively. However, the increase was more pronounced in PP/5GNP compared with PP/5nTiO_2_. A comparison between different types of fillers at 5 wt % loading showed that the GNP composite gained higher flexural strength and flexural modulus properties compared with SCF and nTiO_2_.

PP/10SCF/GNP hybrid composites at different wt % GNP loadings showed an improvement in flexural modulus and flexural strength with the addition of wt % GNP loading. However, in the case of PP/10SCF/nTiO_2_ hybrid composites, the flexural modulus and flexural strength improved but not as pronounced as in PP/10SCF/GNP.

**Figure 13:**
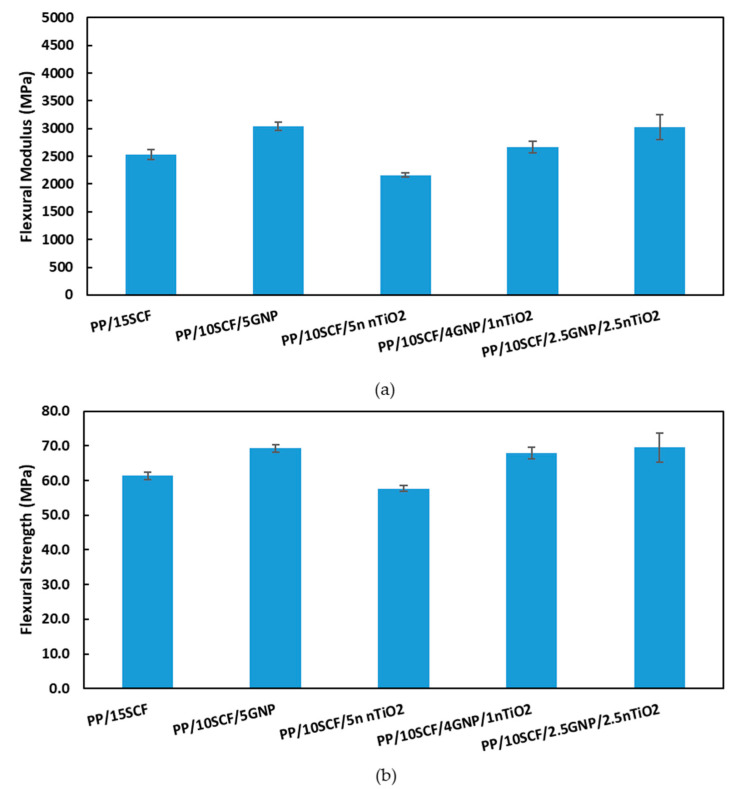
(**a**) Flexural modulus and (**b**) flexural strength at 15% loading for different composites.

Figure 13a,b shows a comparison of flexural modulus and flexural strength at 15 wt % loading for different composites. At 15 wt % loading for different composites, the highest flexural strength and flexural modulus were for PP/10SCF/5GNP and PP/10SCF/2.5GNP/2.5nTiO_2_ hybrid composites, which increased by ~200% and ~87%, respectively, when compared with the neat PP. In general, hybrid composites have a higher flexural strength and flexural modulus compared with PP/SCF composites at the same wt % loading.

### 3.8. Impact Fracture Surfaces

**Figure 14:**
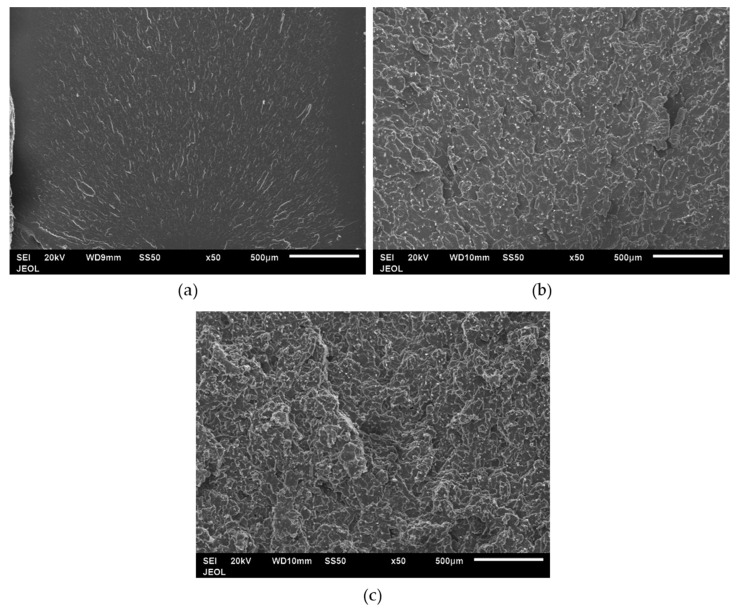
SEM images impact fracture surface of (**a**) neat PP, (**b**) PP/10SCF, and (**c**) PP/20SCF composites.

**Figure 15:**
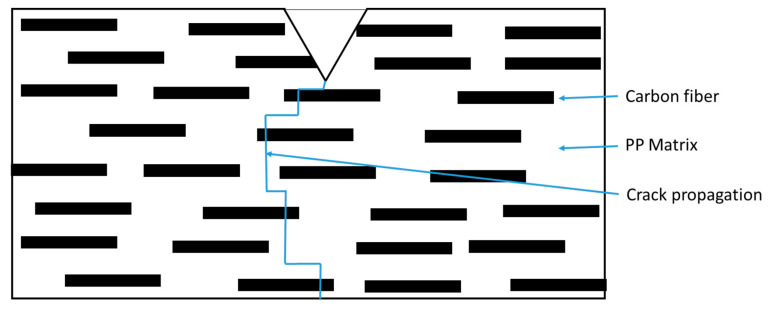
Illustration of impact crack propagation on polypropylene (PP)/short carbon fiber (SCF) composites.

**Figure 16:**
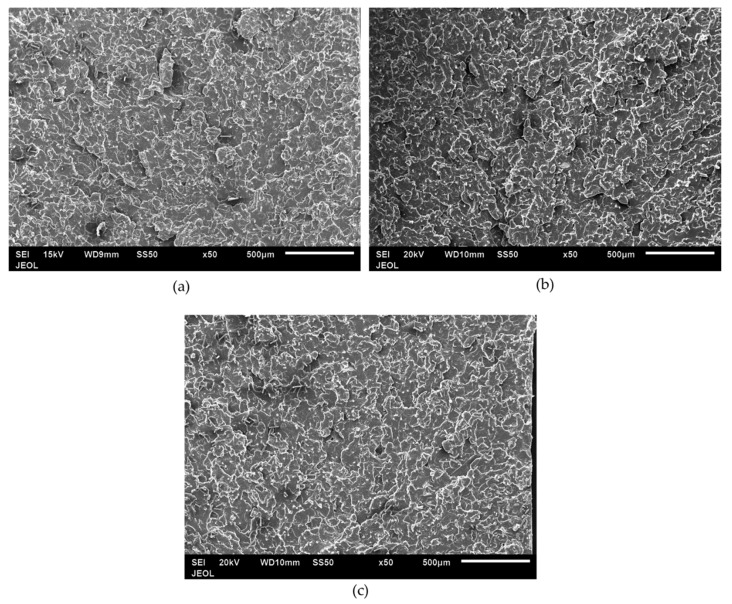
SEM images of impact fracture of hybrid composites of (**a**) PP10SCF/5GNP, (**b**) PP/10SCF/5nTiO_2_, and (**c**) PP/10CF/2.5GNP/2.5nTiO_2_.

Figure 14 shows the impact fracture surface of the PP composite from 0–20 wt % SCF loading. Neat PP in Figure 14a shows a flat fracture surface while the PP/SCF composites show a rough surface. With an increase in the SCF content, there was an increase in the roughness of the fracture surface. Figure 14b,c shows grainy surfaces, which is an area surrounded by white lines. The white lines in the figures show the edges of surfaces of different heights. With the increase in SCF content, the grain became smaller. This means rougher fracture surfaces and implies that the increasing presence of SCF inhibits crack propagation. Cracks were deflected through ends of fiber instead of going into a straight pattern, as illustrated in Figure 15. Figure 16 shows the fracture surfaces of hybrid composites, PP/10SCF/5GNP, PP/10SCF/5nTiO_2_, and PP/10SCF/2.5GNP/2.5nTiO_2_. The figures also show a rougher impact fracture surface compared to neat PP.

### 3.9. Impact Test

Table 7 shows that the impact toughness of the composites increased with the addition of carbon fiber or nanofillers. The maximum impact toughness was 3.6 kJ/m^2^ at 20 wt % loading of SCF, an increase of 75% compared with the impact toughness of the neat PP. The improvement of impact toughness with the addition of SCF could be due to crack deflection as explained before in the impact fracture surface section. In addition, this improvement could also be a result of the fracture mechanism, which involves fiber pull-out and fiber fracture [54].

For the PP/GNP composite system, the presence of GNP increases the impact toughness to 2.7 kJ/m^2^ at 1 and 2.5 wt % loading, from a value of 2 kJ/m^2^ for neat PP. However, additional loading to 5 wt % results in a drop in the impact toughness of the composite. The increase in impact toughness could also be attributed to the bridging effect of GNP [21] and the decrease due to agglomeration and GNP that was oriented parallel to impact fracture surface [47]. Meanwhile, for the PP/nTiO_2_ composite, the increase in nTiO_2_ loading up to 5% resulted in an increase in the impact toughness to 2.6 kJ/m^2^. The nTiO_2_ improves the impact toughness of the composite by crack blunting [48] and plastic void growth [49,50]. The combination of GNP and nTiO_2_ at 5 wt % total loading in PP/GNP/nTiO_2_ composites can improve the impact toughness up to 3 kJ/m^2^ for PP/2.5GNP/2.5nTiO_2_ composites compared to PP/GNP and PP/nTiO_2_ alone. A combination of nanofillers can improve the dispersion of nanofiller in the composite, leading to improved impact toughness [30].

For PP/10SCF/GNP composite systems, a maximum impact toughness of 3.1 kJ/m^2^ was observed at 1 wt % of GNP and then it started to drop with the addition of more GNP. This drop was also noticed in the PP/GNP system. PP/10SCF/nTiO_2_ composites show a gradual increase in impact toughness with the addition of nTiO_2_ up to 5 wt % up to 3.2 kJ/m^2^, which was also observed in PP/nTiO_2_ composites. The presence of nanofillers in PP/SCF composites also improved the impact toughness as observed for PP/nanofillers composites.

**Figure 17:**
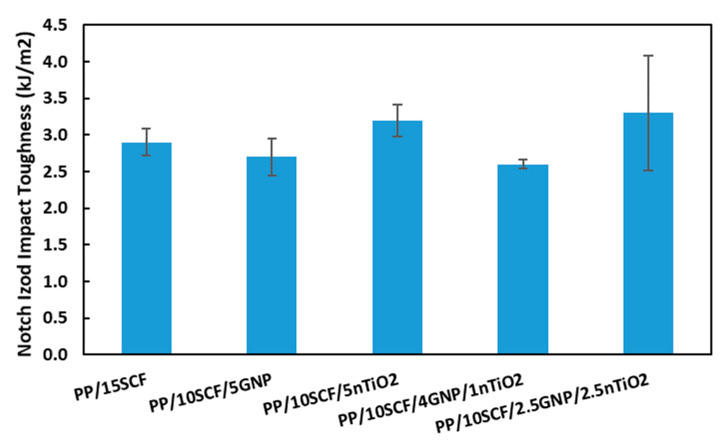
Notch Izod impact toughness at 15 wt % loadings for different composites.

Figure 17 shows the comparison of notch Izod impact toughness at different composites with 15 wt % total loading. It shows that the highest impact toughness was exhibited by the PP/10CF/5nTiO_2_ composite and the PP/10CF/2.5GNP/2.5nTiO_2_ composite with the value of 3.2 kJ/m^2^, which increased by 60% when compared to neat PP and was 10% higher than PP/15SCF. The combination of different nanofillers has the potential to improve the impact toughness.

## 4. Conclusions

PP-based composites were developed with SCF, GNP, and nTiO_2_ and combinations of these fillers. The MFI decreased with the addition of SCF, GNP, and nTiO_2_ and their combinations. However, GNP had more of an effect on MFI than SCF and nTiO_2_ fillers. The density of composites increased compared with neat PP with the addition of SCF and nanofillers. The measured density agreed with the theoretical density. The modulus of elasticity and UTS of the PP/SCF, PP/nanofiller, and PP/SCF/nanofiller composites increased with the addition of wt % of SCF or nanofillers. For PP/SCF and PP/10SCF/nanofillers, the ductility tended to drop significantly. For PP composites at 15 wt % loading with different fillers, PP/10SCF/4GNP/1nTiO_2_ and PP/10SCF/2.5GNP/2.5nTiO_2_ had better UTS and strain at break than the PP/15SCF composite, while the tensile modulus tended to be almost the same. The PP/10CF/2.5GNP/2.5nTiO_2_ hybrid composite was also 20% and 13% higher in flexural strength and flexural modulus, respectively, compared with the PP/15SCF composite. Strain at break for PP/10CF/2.5nTiO_2_ was 15% and 41% higher than for PP/10SCF and PP/15SCF composites, respectively. In general, the notch Izod impact toughness increased with the addition of SCF, GNP, and nTiO_2_ and their combinations compared with neat PP. For a maximum of 15 wt % of filler loading, the highest improvement was shown by the hybrid composite PP/10CF/2.5GNP/2.5nTiO_2_, which improved by 65% and 14% compared with neat PP and the PP/15SCF composite.

## Figures and Tables

**Table 1:** Properties of the raw materials *.

Properties	Unit	PP	SCF	GNP	nTiO_2_
Grade	-	Homopolymer isotactic	PAN-based	Asbury Nano307	Rutile
Density	g/cc	0.9	1.73–1.79	2.16	4.23
Tensile strength	MPa	27.6	2000–3800	-	-
Young’s modulus	GPa	1.5	180–240	-	-
Shape	-	Particulate	Fiber	Sheet/plate	Sphere

* properties are obtained from the manufacturer. PP: polypropylene; SCF: short carbon fiber; GNP: graphite nano-platelet.

**Table 2:** Composition of the polypropylene composites.

Compositions	wt % SCF	wt % GNP	wt % nTiO_2_
Neat PP	0	0	0
PP/5SCF	5	0	0
PP/10SCF	10	0	0
PP/15SCF	15	0	0
PP/20SCF	20	0	0
PP/1GNP	-	1	-
PP/2.5GNP	-	2.5	-
PP/5GNP	-	5	-
PP/1nTiO_2_	-	-	1
PP/2.5nTiO_2_	-	-	2.5
PP/5nTiO_2_	-	-	5
PP/10SCF/1GNP	10	1	-
PP/10SCF/2.5GNP	10	2.5	-
PP/10SCF/5GNP	10	5	-
PP/10SCF/1nTiO_2_	10	-	1
PP/10SCF/2.5nTiO_2_	10	-	2.5
PP/10SCF/5nTiO_2_	10	-	5
PP/4GNP/1nTiO_2_	-	4	1
PP/2.5nTiO_2_/2.5GNP	-	2.5	2.5
PP/10SCF/4GNP/1nTiO_2_	10	4	1
PP/10SCF/2.5GNP/2.5nTiO_2_	10	2.5	2.5

**Table 3:** The surface area of the three fillers: short carbon fiber (SCF) micro-size, graphite nano-platelet (GNP), and Titanium dioxide nanoparticles (nTiO_2_).

Filler	Surface Area/Weight (m^2^/g)	Surface Area/Volume (m^2^/cm^3^)
SCF	0.33	0.59
GNP	350	756
nTiO_2_	48	200

**Table 4:** Average fiber length, as-received, and after loading.

	Average Fiber Length (µm)
As-received	150 ± 100
PP/10SCF	115 ± 49
PP/15SCF	110 ± 60
PP/10SCF/5GNP	86 ± 45
PP/10SCF/5nTiO_2_	107 ± 53
PP/10SCF/2.5GNP/2.5nTiO_2_	87 ± 52

**Table 5:** Melt flow index (MFI), actual density and theoretical density of the polypropylene (PP) composites.

Compositions	MFI (g/10 min)	Actual Density (g/cm^3^)	Theoretical Density (g/cm^3^)
Neat PP	22.7 ± 1.2	0.900	0.900
PP/5SCF	14.5 ± 0.4	0.922	0.923
PP/10SCF	12.7 ± 0.2	0.947	0.947
PP/15SCF	11.3 ± 0.3	0.970	0.973
PP/20SCF	10.1 ± 0.2	0.997	0.999
PP/1GNP	8.9 ± 0.2	0.906	0.905
PP/2.5GNP	7.1 ± 0.1	0.914	0.913
PP/5GNP	6.2 ± 0.1	0.927	0.927
PP/1nTiO_2_	18.3 ± 0.9	0.906	0.907
PP/2.5nTiO_2_	13 ± 0.3	0.918	0.918
PP/5nTiO_2_	9.9 ± 0.3	0.937	0.937
PP/10SCF/1GNP	7.9 ± 0.2	0.954	0.953
PP/10SCF/2.5GNP	5.2 ± 0.1	0.964	0.962
PP/10SCF/5GNP	4.4 ± 0	0.977	0.977
PP/10SCF/1nTiO_2_	10.8 ± 0.1	0.955	0.955
PP/10SCF/2.5nTiO_2_	10.9 ± 0.4	0.966	0.967
PP/10SCF/5nTiO_2_	9.6 ± 0.3	0.988	0.988
PP/4GNP/1nTiO_2_	7.1 ± 0.2	0.929	0.929
PP/2.5nTiO_2_/2.5GNP	6.7 ± 0.1	0.933	0.932
PP/10SCF/4GNP/1nTiO_2_	6.1 ± 0.1	0.979	0.979
PP/10SCF/2.5GNP/2.5nTiO_2_	6 ± 0.2	0.982	0.983

**Table 6:** Tensile modulus, strain at break, and ultimate tensile strength (UTS) of neat polypropylene (PP) and its composites.

Compositions	Tensile Modulus (MPa)	Strain at Break (%)	UTS (MPa)
Neat PP	1358 ± 35	845 ± 26.1	27.6 ± 0.3
PP/5SCF	2170 ± 117	449 ± 34.1	29.4 ± 0.3
PP/10SCF	3456 ± 395	7.8 ± 0.3	34.5 ± 0.3
PP/15SCF	4329 ± 258	6.4 ± 0.3	38.5 ± 0.5
PP/20SCF	6314 ± 83	5.3 ± 0.2	46.9 ± 0.3
PP/1GNP	1871 ± 8	691 ± 74	31.5 ± 0.07
PP/2.5GNP	1886 ± 16	649 ± 23	32.91 ± 0.28
PP/5GNP	2061 ± 1	514 ± 10	33.6 ± 0.14
PP/1nTiO_2_	1504 ± 21	833 ± 23	28.5 ± 0.15
PP/2.5nTiO_2_	1521 ± 30	789 ± 66	28.9 ± 0.42
PP/5nTiO_2_	1643 ± 67	781 ± 18	29.2 ± 0.36
PP/10SCF/1GNP	3622 ± 294	8.7 ± 0.4	38.9 ± 0.34
PP/10SCF/2.5GNP	3968 ± 187	8.7 ± 0.8	39.8 ± 0.59
PP/10SCF/5GNP	4390 ± 97	6.8 ± 0.5	41.5 ± 0.06
PP/10SCF/1nTiO_2_	3776 ± 88	8.3 ± 0.1	38 ± 0.45
PP/10SCF/2.5nTiO_2_	3812 ± 133	9 ± 0.5	38.1 ± 0.27
PP/10SCF/5nTiO_2_	4097 ± 328	8.3 ± 2.3	37.7 ± 0.56
PP/4GNP/1nTiO_2_	2046 ± 36	580 ± 59	32.7 ± 0.6
PP/2.5nTiO_2_/2.5GNP	1963 ± 65	576 ± 84	33.1 ± 0.3
PP/10SCF/4GNP/1nTiO_2_	4417 ± 348	7.7 ± 1	42.3 ± 1.54
PP/10SCF/2.5GNP/2.5nTiO_2_	4229 ± 45	7.3 ± 0.4	41.1 ± 0.1

**Table 7:** Flexural test results and impact toughness of the composites.

Compositions	Flexural Modulus (MPa)	Flexural Strength (MPa)	Impact Toughness (kJ/m^2^)
Neat PP	989 ± 34	37.1 ± 0.7	2 ± 0.06
PP/5SCF	1391 ± 30	42.7 ± 0.5	2 ± 0.0
PP/10SCF	2066 ± 32	54.2 ± 0.7	2.5 ± 0.28
PP/15SCF	2536 ± 88	61.4 ± 1.1	2.9 ± 0.18
PP/20SCF	3369 ± 119	74.4 ± 0.4	3.6 ± 0.13
PP/1GNP	1334 ± 42	43.7 ± 1.3	2.7 ± 0.1
PP/2.5GNP	1443 ± 25	46.2 ± 1	2.7 ± 0.16
PP/5GNP	1578 ± 58	48.6 ± 1.3	2.6 ± 0.14
PP/1nTiO_2_	1036 ± 16	37.7 ± 0.6	2.3 ± 0.24
PP/2.5nTiO_2_	1063 ± 14	38.4 ± 0.8	2.5 ± 0.09
PP/5nTiO_2_	1146 ± 61	40.8 ± 1.7	2.6 ± 0.23
PP/10SCF/1GNP	2461 ± 26	61 ± 0.7	3.1 ± 0.55
PP/10SCF/2.5GNP	2793 ± 107	65.6 ± 1.6	2.6 ± 0.34
PP/10SCF/5GNP	3043 ± 72	69.3 ± 1.1	2.7 ± 0.25
PP/10SCF/1nTiO_2_	2119 ± 272	55.1 ± 1.9	2.7 ± 0.02
PP/10SCF/2.5nTiO_2_	2084 ± 44	55.7 ± 1	2.9 ± 0.12
PP/10SCF/5nTiO_2_	2163 ± 32	57.7 ± 0.9	3.2 ± 0.22
PP/4GNP/1nTiO_2_	1421 ± 31	47.3 ± 0.8	2.6 ± 0.33
PP/2.5nTiO_2_/2.5GNP	1489 ± 67	48.1 ± 1.5	3 ± 0.12
PP/10SCF/4GNP/1nTiO_2_	2663 ± 107	67.9 ± 1.7	2.6 ± 0.06
PP/10SCF/2.5GNP/2.5nTiO_2_	3024 ± 221	69.5 ± 4.2	3.3 ± 0.88
